# Draft genomes of non-nitrogen-fixing* Frankia* strains

**DOI:** 10.7150/jgen.65429

**Published:** 2021-10-22

**Authors:** Camila Carlos-Shanley, Trina Guerra, Dittmar Hahn

**Affiliations:** Texas State University, Department of Biology, 601 University Drive, San Marcos, TX 78666, USA.

**Keywords:** *Frankia*, Actinorhizal symbiosis, genome, non-nitrogen-fixing frankiae, biosynthetic gene clusters

## Abstract

In this study, we describe the genomes of two novel candidate species of non-nitrogen fixing *Frankia* that were isolated from the root nodules of *Coriaria nepalensis* and *Alnus glutinosa*, genospecies CN and Ag, respectively*.* Comparative genomic analyses revealed that both genospecies lack genes essential for nitrogen-fixation and possess genes involved in the degradation of plant cell walls. Additionally, we found distinct biosynthetic gene clusters in each genospecies. The availability of these genomes will contribute to the study of the taxonomy and evolution of actinorhizal symbioses.

## Introduction

The actinobacterial genus *Frankia* consists of a diverse group of filamentous, gram-positive soil bacteria that are able to form root nodules with members of eight plant families representing about 25 genera of woody, dicotyledonous, perennial angiosperms 1-3. Root nodule formation is host plant-specific, and largely correlates with assignments of strains to specific clusters derived from comparative sequence analyses of amplicons of genes such as 16S or 23S rRNA genes 4, *nif*H genes 5-7, *gyr*B or *gln*II 8 and concatenated sequences of some housekeeping genes (*dna*A, *fts*Z, and *pgk*) 9, 10. Clusters 1, 2 and 3 represent *Frankia* strains that are capable of fixing atmospheric nitrogen (N_2_) in pure culture and in symbiosis, while members of cluster 4 are typically unable to fix N_2_, with one exception, and are often not able to form root nodules 4, 11.

While comparative sequence analyses of specific gene amplicons have provided evidence for group-specific assignments of isolates, whole genome sequencing has opened a new avenue to more sophisticated classification of species within the genus *Frankia*. These analyses include both isolates deposited as type strains in culture collections, and uncultured *Frankia* populations in root nodules of specific host plants described as candidate species. *Frankia alni* ACN14a^T^ 12 and *Frankia torreyi* CpI1^T^ 13, as well as *Frankia casuarinae* CcI3^T^ 12 and *Frankia canadensis* ARgP5^T^ 14 have been identified in cluster 1, representing cluster 1a, 1c, and 1d, respectively. Related, but distinct to *F. candadensis* is Candidatus *Frankia nodulisporulans* AgTrS, and AgUmASt1 and AgUmASH1 15 as well as Candidatus *Frankia alpina* AiOr, and AvVan 15. *Frankia coriariae* BMG5.1^T^ 16, Candidatus *Frankia datiscae* Dg1 17, Candidatus *Frankia californiensis* Dg2 18 and Candidatus *Frankia meridionalis* Cppng1 19 represent species in cluster 2, while *Frankia elaeagni* BMG5.12^T^ 12, *Frankia discariae* BCU110501^T^ 20, *Frankia soli* NRRL B-16219^T^ 21 and *Frankia irregularis* G2^T^ 22 are species in cluster 3. Cluster 4 frankiae are represented by three species, *Frankia inefficax* EuI1c^T^ 23, *Frankia asymbiotica* M16386^T^ 24 and *Frankia saprophytica* CN3^T^ 25.

Using comparative sequence analyses of amplicons of an actinobacteria-specific insertion in the 23S rRNA genes of additional cluster 4 frankiae, we identified several strains clustering together but distinct from type strains of cluster 4 26. These strains included CN4, CN6, CN7, and CNM7 isolated from *Coriaria nepalensis* 27, AgW1.1 and AgB1.9 isolated from *Alnus glutinosa* 28, and a yet undescribed strain AgB1.8 obtained from the same location as strain AgB1.9. The goal of this study was to use whole genome sequence analyses to assess the viability of our previous amplicon-based analysis, and thus affirm the potential of these strains for new species descriptions.

## Materials and Methods

### Sample preparation

Seven *Frankia* strains previously identified to represent members of cluster 4 (AgW1.1, AgB1.9, AgB1.8, CN4, CN6, CN7, CNM7) were grown in Defined Propionate Medium (DPM) containing propionate and NH_4_Cl as C and N source, respectively 29, at 30°C for two weeks. Cells were harvested by centrifugation (15,000 x g, 5 min), and cell aggregates homogenized by brief sonication (10s at 20% output in a S-450 sonifier, Branson Ultrasonics, Danbury, CT) 30. After centrifugation, cell pellets were used for DNA extraction using the SurePrep^TM^ Soil DNA Isolation Kit (Fisher Scientific, Houston, TX) with small modifications as described before 31. Extractions of all samples were done in triplicate, and DNA concentrations measured with a Qubit^®^ 2.0 Fluorometer (Life Technologies, Carlsbad, USA). Library preparation and sequencing was performed at the Microbial Genomics Sequencing Center, Pittsburgh, PA, USA using the Illumina tagmentation protocol and the NextSeq Illumina platform (2 x 150 bp).

### Genome assembling

Sequence reads were filtered and trimmed using the default settings of fastp 32, and bbduk (https://jgi.doe.gov/data-and-tools/bbtools/bb-tools-user-guide/) was used to remove reads with average % GC < 54. Genomes were assembled using SPAdes 3.13.0 33. Quast was used to check the quality of the assembled genomes.

### Comparative genomic analysis

All “*Frankia*” genomes available in NCBI Genomes (https://www.ncbi.nlm.nih.gov/genome/) in November, 14, 2019 were downloaded for comparative genomic analyses. Anvi'o 34 was used to compare the shared COG functions among *Frankia* genomes and generate a core genome phylogenetic tree. The core genome phylogenetic tree was generated using anvi-gen-phylogenomic-tree with the concatenated alignment of 113 single-copy core genes (present in all genomes) that resulted in a total alignment length of 40,557 amino acids. Fragments of the 16S rRNA gene were recovered from the genomes using the HMM profile Ribosomal RNAs implemented in anvi'o. 16S rRNA gene sequences were aligned using SINA 35 and sequence identity was calculated with MEGAX 3 36. Pairwise Average Nucleotide Identity (ANI) was calculated with the fastANI software 37. Identification of open reading frames (ORFs) was performed using Prokka using the default settings 38 and KEGG orthologous annotation was performed with kofamKOALA using the default settings 39. We used antiSMASH 5.0 using the default settings 40 to investigate the presence of biosynthetic gene clusters (BGCs) categories across cluster 4 *Frankia* strains.

## Results

### Phylogenetic analysis of *Frankia* spp. isolates

**Figure 1:**
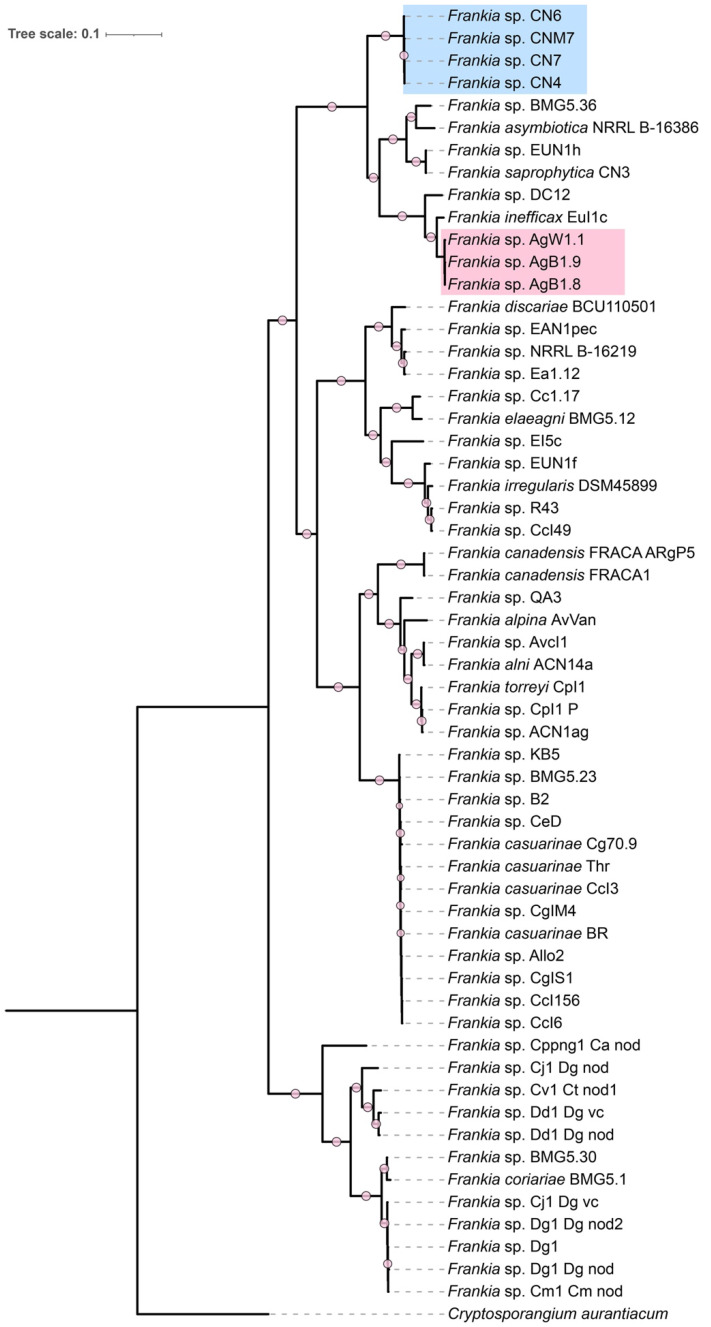
Maximum likelihood tree from concatenated alignments of 113 single-copy protein (total alignment length of 40,557 amino acids) generated with Anvi'o. Strains sequenced in this study are highlighted. The tree was re-rooted using *Cryptoporangium aurantiacum* as outgroup. Circles represent branch support values (maximum likelihood bootstrap) ≥ 0.8.

Assembled genomes ranged from 9.8 Mb and 10.7 Mb and the GC content of the genomes was between 71.5 and 72.7% (Table 1). A phylogenetic tree generated from the concatenated alignment of single-copy orthologous proteins present in all 58 *Frankia* genomes revealed that the closest strains to AgB1.8, AgB1.9, AgW1.1, CNM7, CN4, CN6, CN7 were members of cluster 4, i.e. *Frankia saprophytica* CN3, *Frankia* sp. EUN1h, *Frankia asymbiotica* NRRL B-16386, *Frankia* sp. BMG5.36, *Frankia* sp. DC12 and *Frankia inefficax* EuI1c (Figure 1).

**Figure 2:**
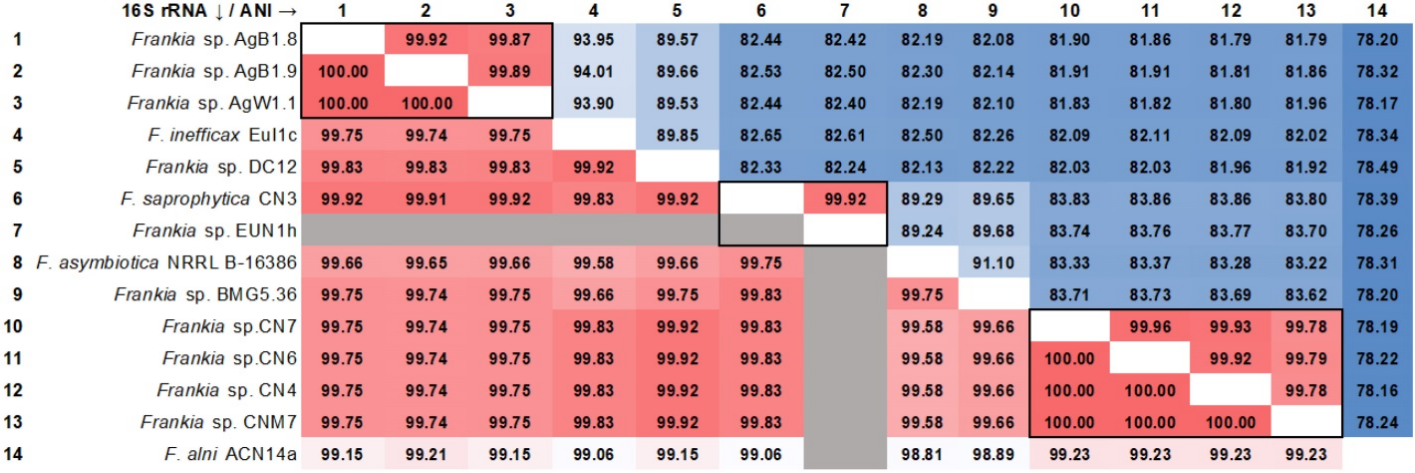
Heatmap matrix of similarity among genomes of cluster 4 frankiae. Top diagonal values correspond to the pairwise Average Nucleotide Identity (ANI) in percentage. Groups of genomes that fall into the estimated species boundary (ANI ≥ 95%) are delimited with black lines. Bottom diagonal values correspond to the pairwise identities of 16S rRNA genes (alignment length 1,202 bp) in percentage. No 16S rRNA sequence of *Frankia* sp. EUN1h was found in the genome using the anvi'o pipeline.

In order to determine if the strains described in this work were potential novel species the average nucleotide identity (ANI) was calculated among all the cluster 4 strains (Figure 2). AgB1.8, AgB1.9, AgW1.1, CNM7, CN4, CN6, CN7 did not show ANI ≥ 95% to any of the reference strains. Strains AgB1.8, AgB1.9, AgW1.1 showed ANI ≥ 99% to each other, which indicates they belong to the same genospecies (genospecies Ag). Strains CNM7, CN4, CN6, CN7 showed ANI ≥ 99% to each other, indicating that they belong to a single genospecies (genospecies CN). Analysis of nearly full length 16S rRNA gene fragment (alignment length 1,202) showed that the identity of cluster 4 frankiae ranged from 99.57% to 100% (Figure 2).

### Functional analysis of *Frankia* spp. isolates

We compared the presence of KEGG orthologues (KO) of the two novel genospecies of *Frankia* Ag and CN with other *Frankia* strains (Table S2). As expected, nitrogenase complex genes (*nif*) were absent in all the seven strains sequenced in this study (Table S2). KEGG orthologues common to all cluster 4 genomes and absent in other frankiae included genes involved in the urea transporter system (K11959 to K11963), and the complete tryptophan metabolism pathway (KEGG module M00038). Additionally, KEGG orthologues involved in the biodegradation of xenobiotics were enriched in cluster 4 *Frankia* genomes when compared to other frankiae (Table S2). 415 KEGG orthologues were found in all 58 *Frankia* genomes analyzed in this study, including: alkaline phosphatases (K01113), which are involved in solubilization of soil phosphate (Nautiyal et al., 2000, Hahn et al., 2003); adherence proteins (K12510 and K12511), described to be involved in *Actinobacillus actinomycetemcomitans* host colonization (Kachalany et al. 2001) (Table S2).

**Figure 3:**
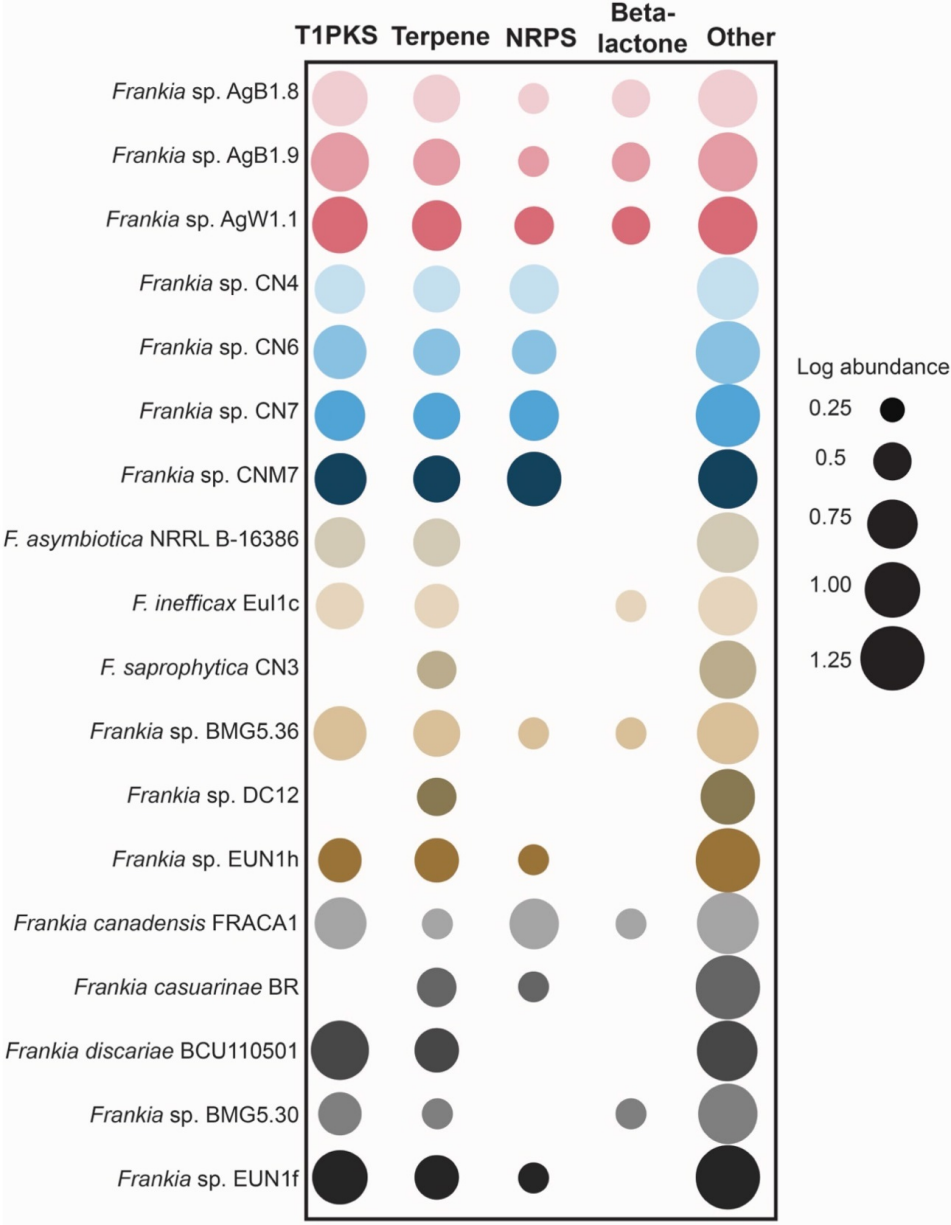
Abundance of biosynthetic gene cluster (BGCs) categories across selected genomes *Frankia* strains. The size of the circles represents the log of total number of clusters of each BGC category in each genome. T1PKS, Type-1 Polyketide synthase; NRPS, Non-ribosomal peptide synthetase.

Unique KOs of genomes of *Frankia* Ag included feruloyl esterase (K09252) and beta-mannosidase (K01192). KOs found in the genomes of *Frankia* CN genospecies and not found in other cluster 4 genomes included a protein involved nitrate transport (K15577), and a flavohemoglobin (K05916). Additionally, genomes of *Frankia* Ag genospecies had more Type I polyketide synthase (PKS) and beta-lactone clusters than other cluster 4 *Frankia*, while *Frankia* CN genospecies had more non-ribosomal peptide synthetase clusters (NRPS) than other cluster 4 frankiae (Figure 3).

## Discussion

In this study, we compared the genomes of three *Frankia* strains isolated from *Alnus glutinosa* and four strains isolated from *Coriaria nepalensis* with 51 publicly available genomes of *Frankia*. Phylogenomic and ANI analyses confirmed that these strains are related to cluster 4 frankiae, and indicate that the three strains isolated from *Alnus glutinosa* (AgB1.8, AgB1.9 and AgW1.1; Ag genospecies) belong to a novel species, as do the four strains from *Coriaria nepalensis* (CN4, CN6, CN7 and CNM7; CN genospecies). Genome sizes of all strains assembled as Ag and CN genospecies were about 9.8 Mb and 10.7 Mb, respectively. Sizes were comparable to those of other strains representing cluster 4 frankiae, i.e. *F. inefficax* and *F. saprophytica* with 8.8 Mb and 10 Mb, respectively, and much larger than genomes of most cluster 1 and 3 frankiae (7.5 Mb to 7.9 Mb), including *F. casuarinae* (4.9 to 5.6 Mb) and *A. nodulisporulans* (4.9 Mb), as well as *F. coriariae* as cluster 2 representative (5.8 Mb) (see 11 for review). An exception was the genome size of *F. irregularis*, a cluster 3 representative with a genome size similar to our cluster frankiae (9.5 Mb) 22, and of related cluster 3 strains R43 (10.44 Mb) 41 and EAN1pec (9.04 Mb) 42.

Smaller genome sizes in frankiae have been related to genome reductions are associated to reduced saprotrophic potential, but not symbiotic potential, while larger genomes created through genome expansions, often through duplication of genes involved in introducing substrates into central metabolic pathways, allowed frankiae to exploit a large variety of environments 17, 42. The comparatively large genomes of cluster 3 and cluster 4 *Frankia* strains therefore suggest an increased saprotrophic potential compared to strains assigned to other clusters. In support of this hypothesis, indigenous cluster 3 frankiae as well as introduced strain EAN1pec have been shown to persist and grow under a broader range of environmental conditions (e.g. plant species, carbon resource or matric potential) than indigenous cluster 1a frankiae or introduced strains ArI3 (cluster 1a) and CcI3 (cluster 1c) 43, 44. The results of these studies have been used to suggest that cluster 3 frankiae represent a group of generalists 44. In addition, cluster 1a frankiae were characteristic populations of later stages in a succession of *Frankia* populations in soil associated with plant growth and succession. Frankiae of cluster 1b represented by *Frankia* strain Ag45/Mut15 were characteristic of soils in early stages of plant-mediated organic matter accumulation. Cluster 1c frankiae represented a group of highly-specialized frankiae that depended on the presence of their host plant to grow saprotrophically in soil 44.

Diversity of frankiae in soils vegetated with host plant species such as *Alnus glutinosa* or non-host plant species such as *Betula nigra* was found to be restricted to cluster 1 and 3 frankiae, and generally members of cluster 1b dominating 26. Cluster 1b frankiae have been detected as major populations in several studies, with absolute numbers depending on the sampling depth, physicochemical conditions and the vegetation 43, 45, 46. So far, cluster 4 frankiae have been detected as major *Frankia* population only in prairie soils where they represented about 50% of all frankiae, that included clusters 1b, 2 and 3, in native, restored and cultivated black prairie soil 26. Native and restored prairie were dominated by *Sorghastrum nutans* (L.) (Indiangrass) and *Andropogon gerardii* Vitman (Big bluestem), while the cultivated prairie site was adjacent to the native prairie site, but cultivated continuously with corn. These results suggest that grasses are supporting a diverse community of frankiae in prairie soils, with significant positive effects on the abundance of cluster 4 frankiae. Although it has been shown that frankiae can grow in the rhizosphere of grasses 47, it is yet unclear for prairie environments whether these effects are related to rhizosphere effects of dense grass root environments or to organic matter accumulation in top soils.

Isolates representing cluster 4 frankiae have been obtained from a variety of different host plant species, i.e. *Coriaria nepalensis*, *Alnus glutinosa*, *Elaeagnus umbellata*, *Datisca cannabina*, and *Ceanothus americanus* (CN3, AgW1.1, EuI1c, DC12 and CaI1) 27, 28, 48-50. Cluster 4 frankiae have also been detected in bioassays highlighting that they form an important fraction of all frankiae in wet soils under *A. glutinosa* 51, 52. Some progenies of *A. glutinosa*, however, exhibited natural resistance to infection by these populations with no or only very small nodules formed 28, 53. The failure to detect nodules has therefore resulted in the assumption that many cluster 4 frankiae are not only ineffective with respect to nitrogen-fixation, but also non-infective with respect to root nodule formation. Cluster 4 frankiae could therefore be characterized as largely or even entirely saprotrophic, with isolates obtained from root nodules as surfaces contaminants 54.

Both genospecies CN and Ag lack genes essential for nitrogen-fixation, as observed for cluster 4 species *F. inefficax* EUI1c^T^ 23 and *F. saprophytica* CN3^T^ 25, but not for *F. asymbiotica* M16386^T^ 24. While *F. inefficax* EUI1c^T^ was observed to form ineffective nodules, none of the other species were able to form nodules on their original host plant. Members of genospecies Ag have been shown to induce small ineffective nodules on their respective host plant *A. glutinosa* 28 while members of genospecies CN failed to induce nodules on their host plant *Coriaria nepalensis* 27. Genomes of genospecies Ag strains harbor genes encoding feruloyl esterases and beta-mannosidases that potentially participate in the degradation of plant cell walls as indicated for some plant fungal pathogens, and could therefore be involved in the plant infection process or in nutrient acquisition 55, 56. Genes for both flavohemoglobin and nitrite/nitrate transport proteins are unique for genospecies CN strains potentially involved in the conversion of nitric oxide to nitrate 57. Our analysis supports previous studies that found significant biosynthetic potential of *Frankia* spp. It has been hypothesized that specialized metabolites, such as the molecules produced by PKS and NRPS gene clusters, might participate in plant-microbial signaling and interactions 58. While our genome studies confirm our previous expectations and affirm the potential of these strains for new species descriptions, the assessment of ecological differences between our genospecies CN and Ag, and described cluster 4 species requires additional experimental data.

## Data Summary

1. Genomes of the strains sequenced in this study from Dr. Dittmar Hahn culture collection and were deposited in the National Center for Biotechnology Information (NCBI), under BioProject Number PRJNA680372. Individual RefSeq assembly accession numbers can be found in Table S1.

2. A list of other *Frankia* genomes utilized in this study can be found in Table S1. All sequences were downloaded from the NCBI Assembly database.

## Supplementary Material

Supplementary tables.

## Figures and Tables

**Table 1:** *Frankia* spp. assembled genomes information.

Strain	RefSeq assembly accessiom	Number of reads ^a^	Number of scaffolds	N50 scaffold ^b^	Total length	GC (%) ^c^	Protein coding genes
AgB1.8	GCF_016792105.1	4,651,340	383	46,557	9,788,199	71.67	9,155
AgB1.9	GCF_016786355.1	6,564,124	278	66,606	9,788,632	71.65	9,075
AgW1.1	GCF_016786345.1	3,546,242	558	35,357	9,961,786	71.53	9,540
CNM7	GCF_016786385.1	3,663,004	1,106	15,926	10,670,374	72.72	10,336
CN4	GCF_016786305.1	5,509,032	607	31,737	10,707,582	72.68	9,988
CN6	GCF_016792085.1	6,112,674	397	48,718	10,719,543	72.69	9,882
CN7	GCF_016786325.1	7,180,944	398	51,919	10,728,937	72.68	9,843

^a^ Number of reads used in the genome assembling after quality control. ^b^ N50 scaffold is the largest length L of which 50% of all nucleotides are in scaffolds of size at least L. ^c^ Total number of G and C nucleotides in the genome, divided by the total length of the genome.
